# The pattern of thyroiditis in multiple sclerosis: a cross-sectional study in a tertiary care hospital in Egypt

**DOI:** 10.1186/s43162-020-00017-w

**Published:** 2020-10-27

**Authors:** Nearmeen M. Rashad, Marwa G. Amer, Waleed M. Reda Ashour, Hassan M. Hassanin

**Affiliations:** 1 Internal Medicine Department, Faculty of Medicine, Zagazig University, Zagazig, Egypt; 2 Clinical Pathology Department, Faculty of Medicine, Zagazig University, Zagazig, Egypt; 3 Neurology Department, Faculty of Medicine, Zagazig University, Zagazig, Egypt

**Keywords:** RRMS, Thyroiditis, Interferon beta-1b, Fingolimod, DMDs

## Abstract

**Background:**

Multiple sclerosis (MS) is an inflammatory demyelinating disease of the central nervous system with varied clinical features. Disease-modifying drugs (DMDs) of MS associated with different types of thyroiditis. In this cross-sectional study, we aimed to assess the prevalence of thyroid dysfunction in MS and to investigate the association between DMDs and the risk of thyroiditis in MS. A cross-sectional study included 100 patients with relapsing-remitting multiple sclerosis (RRMS) in relapse, and the diagnosed was according to revised McDonald’s criteria 2010.

**Results:**

Our results revealed that the prevalence of thyroiditis was 40%; autoimmune (34%) and infective (6%) among patients with RRMS in relapse and cerebellar symptoms were significantly higher in patients with thyroiditis compared to patients without thyroiditis. Regarding the association between DMDs and thyroiditis, the prevalence of patients treated with interferon-beta-1b was higher in MS patients with thyroiditis compared to MS patients without thyroiditis. However, the prevalence of patients treated with interferon-beta-1a was lower in MS patients with thyroiditis compared to MS patients without thyroiditis. In addition, we found CMV infection was more common in patients treated by interferon beta-1b and candida infection was common in patients treated by fingolimod.

**Conclusions:**

Thyroiditis is commonly observed in patients with RRMS in relapse and higher prevalence of patients treated with interferon-beta-1b which is commonly associated with thyroiditis and CMV infection; however, candida thyroid infection was common in MS patients treated by fingolimod.

## Background

Multiple sclerosis (MS) is a chronic inflammatory and neurodegenerative disorder of the central nervous system [1]. MS has variable clinical types: acute fulminate forms, clinically isolated syndrome (CIS), relapsing-remitting MS (RRMS), and secondary chronic progressive (SP) MS [2].

Mounting evidence indicates that MS associated with other autoimmune diseases as they have the same genetic or environmental exposures. In further, as new therapies emerge that raise the risk of autoimmune diseases such as thyroid disease, a growing body of evidence has corroborated that most immune therapies for MS are associated with immunosuppression, both short-term/intermittent (pulsed, induction) and long-term persistent immunosuppression (chronic, maintenance). Immune suppression medication associated with opportunistic or increased infections [3].

Disease-modifying drugs (DMDs) have been utilized to ameliorate MS condition including intramuscular interferon beta-1a, subcutaneous IFN-b, subcutaneous IFNb-1b, and fingolimod [4, 5]. Most of these drugs act on the immune system and suppress immune cells so that auto-reactive immune cells will be unable to attack the myelin sheaths of neurons [6].

Thyroid dysfunction (TD) frequently occurs as an autoimmune complication of immune reconstitution therapy (IRT), especially in individuals with multiple sclerosis treated with DMDs. Moreover, the immune suppression of DMDs is associated with opportunistic or increased infections. We aimed in this study to assess the prevalence of thyroid dysfunction in MS and to investigate the possible causes of thyroiditis in MS in relation to DMDs.

## Methods

This cross-sectional study enrolled 200 consecutive patients fulfilling the diagnostic criteria for MS according to the revised McDonald’s criteria 2010 [7]. The patients were recruited from outpatient clinics of Neurology and Internal Medicine Departments, and 40 age- and sex-matched healthy controls. The MS group were diagnosed with relapsing-remitting multiple sclerosis (during relapse) and were divided into two subgroups according to thyroid autoantibodies. The enrolled patients met all of the following criteria of infectious thyroiditis as painful thyroid palpation, fever, and neck pain, together with laboratory tests indicating acute systemic inflammation and abnormal thyrotropin and free thyroxin levels. The diagnosis of autoimmune thyroiditis was obtained based on clinical findings and positive serum antibodies; anti-thyroglobulin antibodies (anti-TG) and anti-thyroid peroxidase antibodies (anti-TPO). All subjects were subjected to thorough history taking and complete general and neurological examination.

Exclusion criteria included pregnancy, lactation, heart, liver, renal, or other endocrine diseases. The diagnosis was supported by thyroid ultrasonographic examination with a 7.5-MHz transducer, and brain and spine MRI were done for all included patients. Laboratory tests were conducted in the Microbiology Unit of Clinical Pathology Department.

### Ethics approval and consent to participate

Written informed consent was taken from all of the participants after explaining the details and benefits as well as risks to them. The ethical committee of the Faculties of Medicine approved the current study.

### Blood sampling and laboratory tests

Blood samples were drawn from all subjects after an overnight fast. Serum C-reactive protein (CRP) was measured using automated clinical chemistry analyzer Cobas Integra 400 plus (Roche Diagnostics, Deutschland). Serum TSH, FT3, and FT4 levels were measured using the Cobas e 601® analyzer (Roche Diagnostics, Mannheim, Germany) by ECLIA. Measurements of serum anti-TG and anti-TPO levels were done by enzyme-linked immunosorbent assay (ELISA) (Genesis Diagnostics, Little port, UK). Complete blood count was done for patients and control using automated hematology analyzer sysmex KX-21N (Sysmex, America).

Blood cultures were done using Bact/ALERT culture bottles and incubated for 7 days in the automated Bact/ALERT 3D Microbial Detection System (bioMérieux, Inc, Durham, USA). The positive blood culture bottles and other isolated samples were initially grown on blood agar and Sabouraud dextrose agar for 24 to 48 h at 37 °C.

### Identification

Candida colonies appeared as flat, smooth, and pale off-white colored and identified by gram stain. Germ tube formation had been tested for the isolated colony to differentiate *C. albicans* against non-albicans candida (NAC). Candida species were identified using matrix-assisted laser desorption ionization-time of flight mass spectrometry (MALDI-TOF MS) (bioMérieux, Marcy l’Etoile, France) according to the manufacturer instructions.

### Antifungal susceptibility testing

The susceptibility to antifungal agents was carried out using Vitek 2 system (bioMérieux, Inc, Durham, USA) for yeast (card no ACT/YS07) containing serial twofold dilutions of six antifungal drugs: fluconazole, flucytosine, voriconazole, caspofungin, micafungin, and amphotericin B were provided by the manufacturer and in accordance with the guidelines.

### Detection of cytomegaly virus (CMV) serological markers

Serological screening of blood samples was performed by detection of anti-CMV IgM and IgG by electro-chemiluminescence immunoassay (ECLIA) technique using Cobas e 410 analyzers (Roche Diagnostics, Mannheim, Germany). All assays were performed according to the recommended manufacturers’ instructions. A reactive result is a sample/cutoff value ≥ 1 for CMV-IgG and > 1 for CMV-IgM.

### Statistical analysis

Statistical analyses were performed using the Statistical Package for the Social Sciences for Windows (version 21.0; SPSS, Chicago, IL, USA). Data were expressed using descriptive statistic (mean ± standard deviation) and were analyzed; the comparison between two groups with parametric variables was done using independent sample *t* test (*t*) and nonparametric variables using Mann–Whitney test (*z*). Logistic regression analysis was performed to determine the variable associated with DMDs. We considered *P* to be significant at < 0.05 with a 95% confidence interval (CI).

## Results

### Clinical and laboratory characteristics of patients with MS

**Fig. 1:**
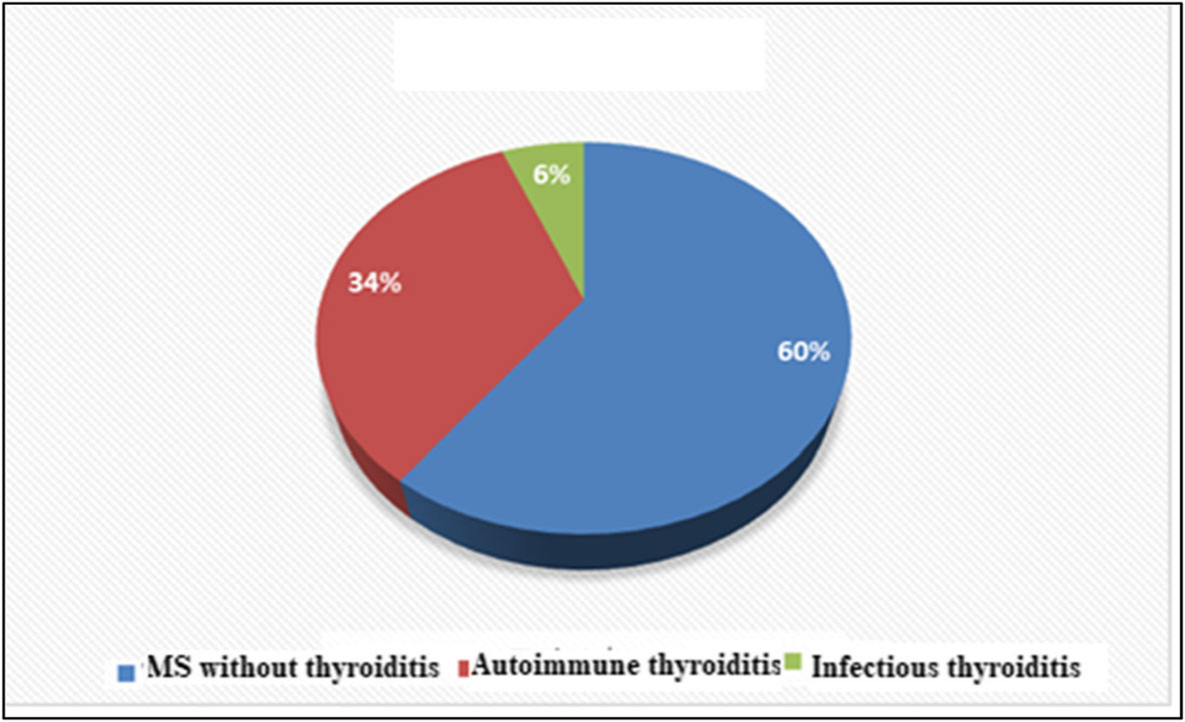
The prevalence of thyroiditis among patients with multiple sclerosis

This cross-sectional study is conducted on patients with RRMS in relapsing. The prevalence of thyroiditis was 40% (autoimmune = 34% and infective = 6%) as shown in Fig. 1. As regards the clinical picture of MS, only cerebellar symptoms were significantly higher in patients with thyroiditis compared to patients without thyroiditis. Patients with thyroiditis as expected had significantly higher values of thyroid autoantibody, anti-TPO, and anti-TG, as well as TSH, compared to MS without thyroiditis. Regarding inflammatory markers, WBC count, neutrophil count, and hs-CRP were significantly higher in the thyroiditis group as compared to MS without thyroiditis. On the contrary, patients with thyroiditis had significantly lower levels of FT3, FT4, hemoglobin, and platelet compared to MS without thyroiditis (Table 1, *P* < 0.05).

**Table 1:** Clinical and laboratory characteristics of patients with MS

Characteristics	MS without thyroiditis (*n* = 120)	MS with thyroiditis (*n* = 80)	*P*
Age (years)	30.81 ± 6.8	30.4 ± 4.98	0.740
Disease duration/year	5.97 ± 0.89	6.45 ± 0.91	˂ 0.05^*^
Number of relapses in the last 2 years	2.97 ± 0.92	3.45 ± 0.93	˂ 0.05^*^
Clinical picture of MS			
Sensory	50 (41.7%)	46 (57.5%)	0.089
Motor	80 (66.7%)	56 (70%)	0.450
Cerebellar	56 (46.7%)	54 (67.5%)	˂ 0.05*
Speech	16 (13.5%)	14 (17.5%)	0.383
Visual	78 (65%)	58 (72.5%)	0.286
Fasting plasma glucose (mg/dl)	87.3 ± 4.8	88.5 ± 3.9	0.732
FT3 (pg/ml)	2.58 ± 0.32	1.01 ± 0.79	˂ 0.001^*^
FT4 (ng/dl)	1.28 ± 0.315	1.11 ± 0.77	˂ 0.001^*^
TSH (μIU/ml)	3.64 ± 1.76	4.27 ± 3.420	˂ 0.001^*^
Anti-TPO (IU/ml)	32.64 ± 8.1	144.87 ± 55.1	˂ 0.001^*^
Anti-TG (IU/ml)	1.1 ± 0.2	1.8 ± 1.52	0.363
WBC count (cell × 10^3^/μl)	10.3 ± 1.1	13.7 ± 6.4	˂ 0.001*
Hemoglobin (g/dl)	9.6 ± 2.71^#^	8.5 ± 1.56	˂ 0.001*
Platelet (cell × 10^3^/μl)	114.8 ± 30.1	77.4 ± 46.7	˂ 0.001^*^
Neutrophil count (cell × 10^3^/μl)	5.61 ± 0.72	10.2 ± 2.68	˂ 0.001*
hs-CRP (μg/ml)	1.16 ± 0.63	5.94 ± 3.89	˂ 0.001^*^
DMDs			
Methylprednisolone	60 (100%)	80 (100%)	–
Interferon beta-1a	18 (30%)	8 (10 %)	˂ 0.001*
Interferon beta-1b	39 (65%)	60 (75.5%)	˂ 0.001*
Fingolimod	14 (23.3%)	12 (15%)	0.224

*TSH* thyroid-stimulating hormone, *FT3* free triidothyronine, *FT4* free thyroxine, *anti-TG* anti-thyroglobulin antibodies, *anti-TPO* anti-thyroid peroxidase antibodies, *WBC* white blood cell, *CRP* C-reactive protein, *DMDs* disease-modifying drugs

*P < 0.05

Regarding the prevalence of DMDs in studied groups, we observed a significantly higher prevalence of MS patients with thyroiditis treated with interferon-beta-1b. However, there was a significantly higher prevalence of MS patients without thyroiditis treated with interferon beta-1a (Table 1, *P* < 0.05).

### Clinical and laboratory characteristics of patients with MS classified according to thyroid function tests

Among the thyroiditis group, the 68 patients had autoimmune and the 12 patients had infective thyroiditis. There were statistically significant increases of anti-TPO, TSH, hemoglobin, and platelet in autoimmune group compared to the infective thyroiditis group (*P* < 0.05). On the other hand, there were significantly lower values of FT3, FT4, WBC count, neutrophil count, and hs-CRP in autoimmune group to infective thyroiditis group of patients (*P* < 0.05) (Table 2).

**Table 2:** Clinical and laboratory characteristics of patients with MS classified according to thyroid function tests

	Autoimmune thyroiditis (*n* = 68)	Infectious thyroiditis (*n* = 12)	*P* value
Age (years)	29.2 ± 4.36	31.6 ± 6.32	0.299
Disease duration/year	6.3 ± 0.96	6.7 ± 0.88	0.215
Number of relapses in the last 2 years	3.3 ± 0.98	3.7 ± 0.87	0.223
Clinical picture of MS			
Sensory	41 (60.2%)	7 (58.3%)	0.580
Motor	51 (76.4%)	8 (66.7%)	0.430
Cerebellar	52 (75%)	7 (58.3%)	0.246
Speech	10 (14.7%)	3 (25%)	0.356
Visual	51 (75%)	9 (75%)	0.645
FT3 (pg/ml)	1.64 ± 0.585	2. 7 ± 0.758	˂ 0.001*
FT4 (ng/dl)	1.3 ± 0.57	2.5 ± 0.75	˂ 0.001*
TSH (μIU/ml)	6.2 ± 1.5	0.133 ± 0.056	˂ 0.001*
Anti-TPO (IU/ml)	193.3 ± 71.08	32.39 ± 5.76	˂ 0.001*
Anti-TG (IU/ml)	1.9 ± 1.49	1.48 ± 1.35	0.337
WBC count (cell × 10^3^/μl)	12.3 ± 1.9	15.7 ± 3.4	˂ 0.001*
Hemoglobin (g/dl)	8.6 ± 2.71	8.5 ± 1.56	0.135
Platelet (cell × 10^3^/μl)	115.8 ± 28.1	34.4 ± 15.1	˂ 0.001*
Neutrophil count (cell × 10^3^/μl)	9.61 ± 1.091	12.12 ± 2.68	˂ 0.001*
hs-CRP (μg/ml)	4.46 ± 1.53	9.64 ± 2.99	˂ 0.001*
DMDs			
Methylprednisolone	68 (100%)	12 (100%)	–
Interferon beta-1a	6 (8.8%)	2 (16.7%)	0.346
Interferon beta-1b	56 (82.3%)	4 (33.3%)	˂ 0.001*
Fingolimod	4 (5.8%)	8 (66.6%)	˂ 0.001*

*TSH* thyroid-stimulating hormone, *FT3* free triidothyronine, *FT4* free thyroxine, *anti-TG* anti thyroglobulin antibodies, *anti-TPO* anti-thyroid peroxidase antibodies, *WBC* white blood cell, *CRP* C-reactive protein, *DMDs* disease-modifying drugs

*P < 0.05

Regarding the prevalence of DMDs in the thyroiditis group, we observed a significantly higher prevalence of autoimmune thyroiditis in patients treated with interferon beta-1b. However, there was a significantly higher prevalence of infective thyroiditis in patients treated with fingolimod (Table 2, *P* < 0.05).

### Prevalence of microorganisms in infective thyroiditis

**Fig. 2:**
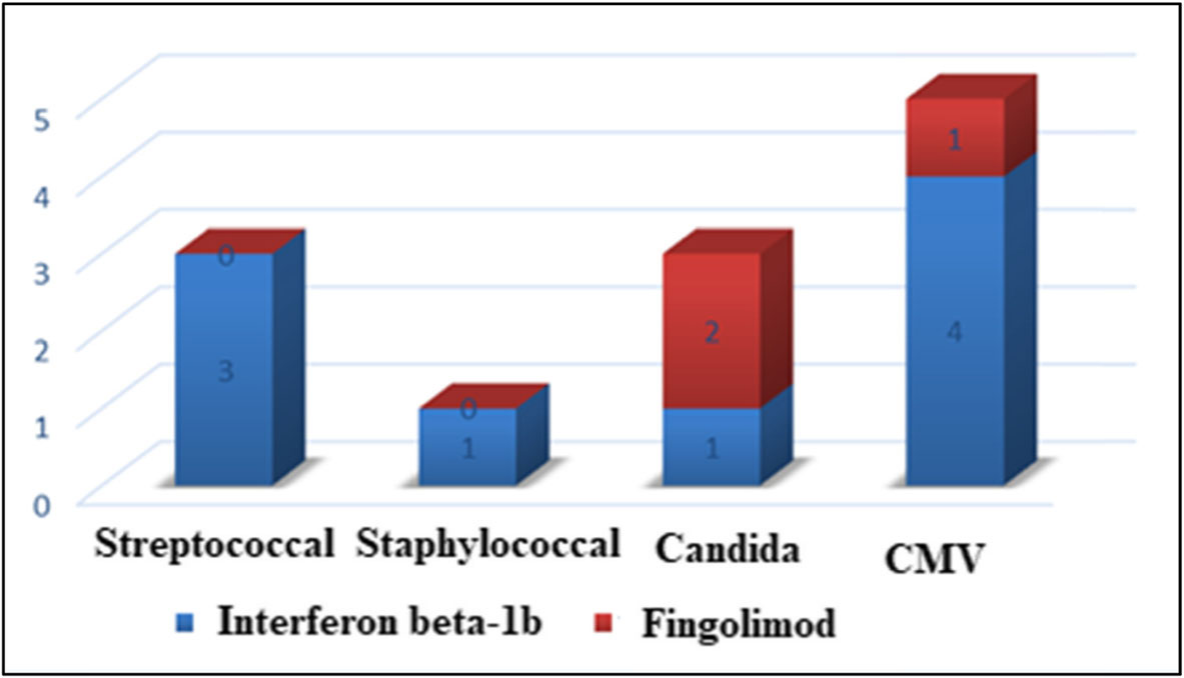
The prevalence of microorganisms in infective thyroiditis

Among infective thyroiditis, 5 patients had CMV, 3 had streptococcal infections, 3 had candida, and only 1 patient had staphylococcal infections in Fig. 2.

### The association between DMDs and types of microorganisms

Regarding the association between DMDs and types of microorganisms among infective thyroiditis groups, we found CMV infection was more common in patients treated by interferon beta-1b and candida infection was common in patients treated by fingolimod (Fig. 2).

### The association between DMDs and levels of thyroid autoantibodies

**Fig. 3:**
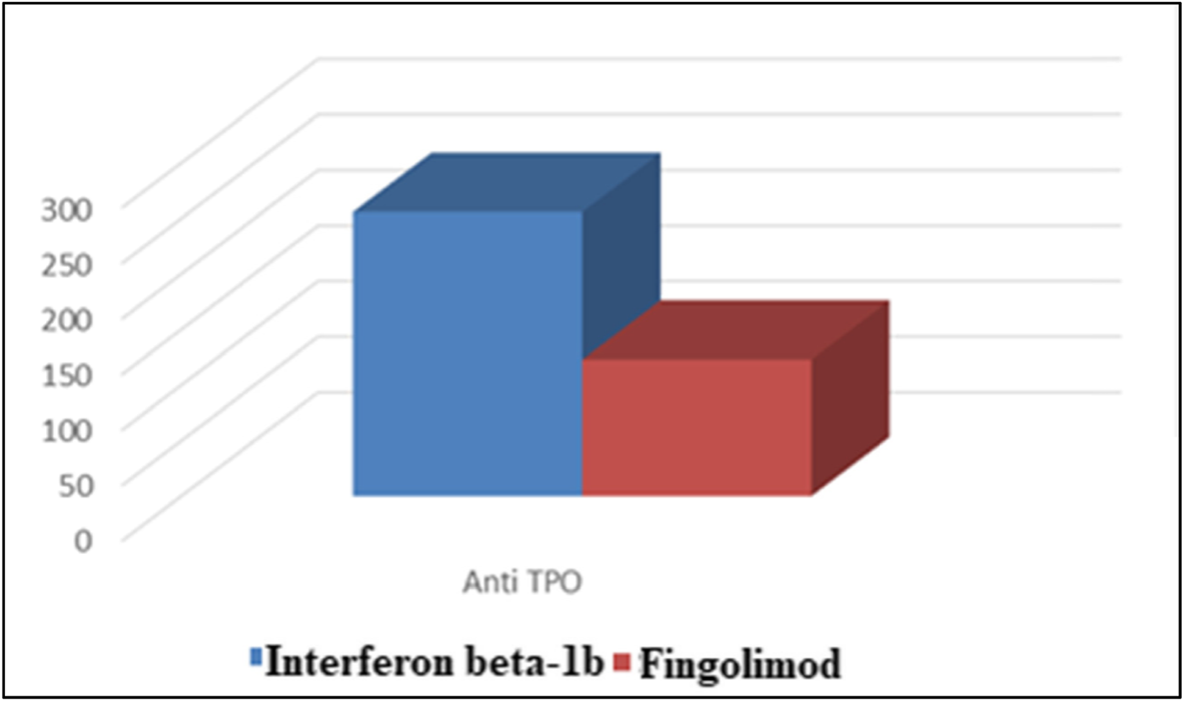
The association between DMDs and levels of anti-TPO

**Fig. 4:**
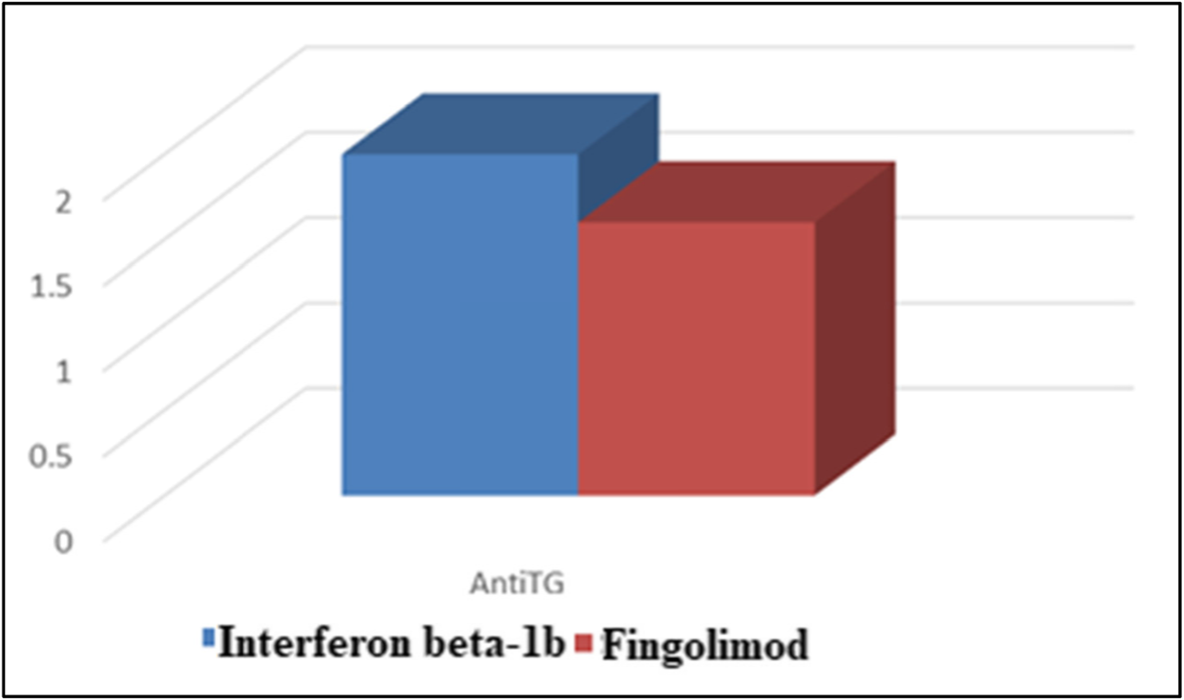
The association between DMDs and levels of anti-TG

Among the autoimmune thyroiditis group, we found the levels of anti-TPO and anti-TG were significantly higher in patients treated by interferon beta-1b compared to patients treated by fingolimod (Figs. 3 and 4, respectively).

### Logistic regression analysis to determine the variable associated with DMDs in MS patients with thyroiditis

We further test our results by a logistic regression test. We observed that FT4, TSH, anti-TPO, anti-TG, WBC count, platelet, neutrophil count, and hs-CRP were significantly associated with DMD (Table 3, *P* < 0.05).

**Table 3:** Logistic regression analysis to determine the variable associated with DMDs in MS patients with thyroiditis

Model		Unstandardized coefficients		Standardized coefficients	*t*	Sig.	95.0% confidence interval for B	
		*B*	Std. error	Beta			Lower bound	Upper bound
1	(Constant)	0.456	0.085		5.368	˂ 0.001*	0.287	0.625
	FT3	− 0.056	0.033	0.066	− 1.707	0.091	− 0.121	0.009
	FT4	0.180	0.032	0.211	5.613	˂ 0.001*	0.116	0.243
	TSH	− 0.068	0.010	− 0.295	− 6.687	˂ 0.001*	− 0.088	− 0.048
	Anti-TPO	− 0.004	0.001	− 0.658	− 5.302	˂ 0.001*	− 0.005	− 0.002
	Anti-TG	− 0.037	0.014	− 0.093	− 2.540	˂ 0.05*	− 0.065	− 0.008
	WBC count	− 0.003	0.001	− 0.147	− 4.944	˂ 0.001*	− 0.004	− 0.002
	Hemoglobin	− 0.017	0.010	− 0.075	− 1.741	0.085	− 0.037	0.002
	Platelet	− 0.005	0.001	− 0.367	− 7.098	˂ 0.001*	− 0.006	− 0.003
	Neutrophil count	0.023	0.006	0.143	3.663	˂ 0.001*	0.011	0.036
	hs-CRP	0.021	0.007	0.135	2.926	˂ 0.05*	0.007	0.036

*TSH* thyroid-stimulating hormone, *FT3* free triidothyronine, *FT4* free thyroxine, *anti-TG* anti-thyroglobulin antibodies, *anti-TPO* anti-thyroid peroxidase antibodies, *TRAb* thyroid receptor antibody, *WBC* white blood cell, *CRP* C-reactive protein, *DMDs* disease-modifying drugs

*P < 0.05

## Discussion

MS is the most prevalent chronic neuroinflammatory disease among young adults and carries the potential risk of permanent disability [8].

Gathering studies have reported the co-occurrence of autoimmune disease with MS, e.g., thyroiditis. As new therapies emerge, the risk of autoimmune diseases rises. We aimed in this study to assess the prevalence of thyroid dysfunction in MS and to investigate the possible causes of thyroiditis in MS in relation to DMDs.

The interesting result of our study was that the prevalence of thyroiditis was 40%, autoimmune 34%, and infective 6% among patients with RRMS in relapse. The current study revealed that cerebellar symptoms were significantly higher in patients with thyroiditis compared to patients without thyroiditis. Regarding inflammatory markers, WBC count, neutrophil count, and hs-CRP were significantly higher in the thyroiditis group as compared to MS without thyroiditis. Similar to our study, Barone et al. revealed that 12.3% of patients with MS had thyroid disease [9].

Against our findings, the results of Marrie et al. observed no difference between the prevalence of thyroid dysfunction and anti-thyroid antibodies in MS patients compared with a control population [10].

Numerous immunosuppressant therapies are used for MS treatment. Overall, these agents are safe, have favorable risk-benefit profiles, and can dramatically improve the quality of life for patients with a potentially disabling neurologic illness, but iatrogenic complications were observed. According to the current study, we observed a significantly higher prevalence of MS patients with thyroiditis treated with interferon-beta-1b. However, there was a significantly higher prevalence of MS patients without thyroiditis treated with interferon beta-1a. We further test our results by a logistic regression test. We observed that FT3, FT4, TSH, anti-TPO, and anti-TG were significantly associated with interferon beta-1b.

Severa et al. reported that IFN-β is a key molecule in multiple sclerosis, as it maintains the anti-inflammatory status of the immune system and is one of the most widely used treatments for MS. As IFN-β modulates the immune-regulatory system, it may precipitate autoimmune disorders. IFN-β therapy has been associated with a relatively high risk of developing thyroid disease, either organ dysfunction or autoimmunity [6].

The thyroid gland is a curiously infection-resistant organ because of its high vascularity, lymphatic drainage, tissue uptake of iodine or hydrogen peroxide, and encapsulated structure. Among the factors that may predispose to infective thyroiditis is an immunocompromised status [11]. In the present study, we observed that 4% of patients with MS had infectious thyroiditis.

The results presented herein are innovative; as this study performs an evaluation of the etiology of infectious thyroiditis in relation to DMDs, we found CMV infection was more common in patients treated by interferon beta-1b and candida infection was common in patients treated by fingolimod.

Jacobs et al. reported that the most common causative organisms of infective thyroiditis are Staphylococci and Streptococci species. Many other organisms such as Acinetobacter, Mycobacterium, Coccidioides, Pseudomonas, Eikenella, Clostridium, Nocardia, *Pneumocystis carnie*, Haemophilus, and Candida have been isolated, but mostly associated with immunosuppression [12].

Oka et al. detected infection with CMV associated with hyperthyroidism after transplantation due to a reversed imbalance in helper/suppressor T cell populations and B cell dysregulation [13]. Similar results observed by Maawali et al. regarding the cause of infective thyroiditis could be due to CMV [14].

Chun et al. suggested that fingolimod induced a dose-dependent reduction in the peripheral lymphocyte counts to 20–30% of the baseline value and it also increases the risk of infections [15].

## Conclusion

Thyroiditis is commonly observed in patients with RRMS in relapse and there was higher prevalence of patients treated with interferon-beta-1b which is commonly associated with autoimmune thyroiditis and CMV infection; however, candida thyroid infection was common in MS patients treated by fingolimod.

## Data Availability

The data that support the findings of this study are available from the corresponding author (nrashad78@yahoo.com) upon reasonable request.

## Abbreviations

MS: Multiple sclerosis
RRM: Relapsing-remitting multiple sclerosis
DMDs: Disease-modifying drugs
CIS: Clinically isolated syndrome
SP: Secondary chronic progressive
IFN b-1a: Interferon beta-1a
TD: Thyroid dysfunction
IRT: Immune reconstitution therapy
Anti-TG: Globulin antibodies
Anti-TPO: Anti-thyroid peroxidase antibodies
FPG: Fasting plasma glucose
CRP: Serum C-reactive protein
CMV: Cytomegaly virus
TSH: Thyroid-stimulating hormone
FT3: Free triiodothyronine
FT4: Free thyroxine
NAC: Non-albicans candida
